# Activation of the S100A8/A9 Alarmin Amplifies Inflammatory Pathways in Equine Ascending Placentitis

**DOI:** 10.3390/ijms27031550

**Published:** 2026-02-04

**Authors:** Kirsten E. Scoggin, Shimaa I. Rakha, Ahmed M. Abdellatif, Fatma Adlan, Yosra A. Helmy, Rebecca Ruby, Barry Ball, Yatta Boakari, Hossam El-Sheikh Ali

**Affiliations:** 1 Maxwell H. Gluck Equine Research Center, Department of Veterinary Science, University of Kentucky, Lexington, KY 40546, USA; kirsten.scoggin@uky.edu (K.E.S.); yosra.helmy@uky.edu (Y.A.H.); b.a.ball@uky.edu (B.B.); 2 Department of Theriogenology, Faculty of Veterinary Medicine, Mansoura University, Mansoura 35516, Egypt; shimaa_ibrahim@mans.edu.eg (S.I.R.); fatmaadlan83@mans.edu.eg (F.A.); 3 Department of Anatomy and Embryology, Faculty of Veterinary Medicine, Mansoura University, Mansoura 35516, Egypt; abdellatif_ma@mans.edu.eg; 4 Veterinary Diagnostic Laboratory, University of Kentucky, Lexington, KY 40511, USA; reru228@uky.edu; 5 Department of Large Animal Clinical Sciences, College of Veterinary Medicine and Biomedical Sciences, Texas A & M University, College Station, TX 77843, USA; yboakari@tamu.edu

**Keywords:** alarmin S100A8/S100A9, calgranulins, chorioallantois, equine, placentitis, S100 proteins

## Abstract

Ascending placentitis is a significant cause of equine pregnancy loss, yet the upstream inflammatory triggers are poorly defined. Recently, we identified S100A8/S100A9 (S100A8/A9) alarmins as potential upstream regulators in a chronic equine placentitis model. The current study aimed to determine whether this upregulation is sustained in the acute model and in clinical cases, and to elucidate the expression of their downstream inflammatory mediators. Using an experimental model, we quantified *S100A8/A9* mRNA expression in acute (*n* = 5) and chronic (*n* = 6) placentitis induced by *Streptococcus equi* ssp. *zooepidemicus*. We found mRNA expression of *S100A8* and *S100A9* was significantly upregulated in chorioallantois during both acute (*p* < 0.001) and chronic (*p* < 0.0001) disease compared to controls (*n* = 5), demonstrating their role is not limited to chronic pathology. A strong positive correlation (*r* = 0.945) underscored their coordinated expression. Immunohistochemistry revealed minimal staining in controls but dense infiltrations of S100A8/A9-positive neutrophils and macrophages in placentitis tissues. To define the clinical relevance of the downstream pathway, we analyzed RNA sequencing data from clinical placentitis cases (placentitis, *n* = 4) compared to normal postpartum placenta (control, *n* = 4). This confirmed upregulation of *S100A8/A9* and revealed a concurrent increase in their receptors (*TLR4*, *RAGE*) and a spectrum of NF-κB-driven effectors, including pro-inflammatory cytokines (*IL1β*, *IL6*, *TNF*), chemokines (*CXCL8*, *CCL2*, *CXCL10*), and the apoptotic mediator *CASP3*. Our findings establish that S100A8/A9 upregulation is a sustained feature of equine placentitis and delineates a coherent S100A8/A9-TLR4/RAGE-NF-κB signaling axis that drives inflammation and tissue damage in clinical disease. These findings highlight the diagnostic potential of S100A8/A9 and position this alarmin system as a promising therapeutic target for mitigating infection-induced pregnancy loss.

## 1. Introduction

Ascending placentitis is the leading cause of late-term pregnancy loss in mares, leading to economic losses to the equine industry. The condition typically arises from an ascending bacterial infection, often with *Streptococcus equi* ssp. *zooepidemicus*, which triggers a profound inflammatory response at the maternal–fetal interface. This response is characterized by a massive recruitment of leukocytes, dysregulation of immune cell population—including heightened helper T cell populations (Th1/Th17/Th2) and diminished regulatory T cells (Treg) response—and a cascade of inflammatory mediators that disrupts placental function, ultimately leading to pregnancy loss [1,2]. Current therapeutic strategies, often initiated after the onset of clinical signs are of limited efficacy, underscoring the critical need to identify upstream regulators of the placental inflammatory cascade to enable targeted intervention.

In equine placentitis, the inflammatory response and endocrine profiles differ between acute and chronic presentations. Acute placentitis often triggers a rapid decline in maternal immunoreactive progesterone (ir-progesterone) concentrations, frequently resulting in abortion within days. In contrast, chronic placentitis is associated with a sustained inflammatory process and a significant increase in ir-progesterone and specific progestagens, such as 5α-DHP and 20α-5P, reflecting fetal stress and altered placental steroid metabolism. While acute cases often lead to fetal loss, chronic cases often lead to the birth of premature foals or stillbirth, highlighting distinct pathophysiological and endocrine pathways between the two conditions [3,4,5].

Among the most potent initiators and amplifiers of inflammation are damage-associated molecular pattern molecules (DAMPs), also known as alarmins. The S100A8/S100A9 (S100A8/A9) heterodimer, also known as calprotectin, is a prototypic alarmin that is released by activated neutrophils and macrophages at sites of inflammation [6,7,8]. S100A8/A9 functions by engaging Toll-like receptor 4 (TLR4) and the receptor for advanced glycation end products (RAGE), leading to Nuclear factor kappa B (NF-κB) activation and the subsequent production of a panel of pro-inflammatory factors, including tumor necrosis factor (TNF), Interleukin-1β (IL1β), Interleukin-6 (IL6), and C-X-C motif chemokine ligand 8 (CXCL8 or IL8) [6,9,10,11,12,13,14]. This alarmin system is critically implicated in reproductive pathology; elevated levels of calgranulin A (S100A8) and calgranulin B (S100A9) have been detected in the placenta and serum of women with recurrent pregnancy loss and intra-amniotic infection [15,16,17,18,19], positioning them as key suspects in inflammatory pregnancy disorders. Beyond reproductive biology, S100A8/A9 is increasingly recognized as a multivalent alarmin with pathogenic roles across a wide spectrum of inflammatory and degenerative diseases. Dysregulated S100A8/A9 signaling has been implicated in cardiovascular disease [20], where it contributes to endothelial activation, vascular inflammation, and atherosclerotic progression, as well as in neurological disorders characterized by chronic neuroinflammation and disruption of immune homeostasis [21,22]. Additionally, emerging evidence supports a role for S100A8/A9 in cancer progression, including modulation of the tumor microenvironment and promotion of metastatic niche formation [10]. Collectively, these findings position S100A8/A9 as a conserved amplifier of innate immune responses across organ systems, highlighting the importance of understanding its context-specific regulation and downstream effects in specialized tissues such as the placenta.

Our previous transcriptomic analysis of equine chronic placentitis uniquely identified S100A8 and S100A9 as potential upstream regulators, as they were exclusively expressed in the placentitis group and absent in controls [23]. Therefore, we hypothesize that S100A8/A9 alarmins are upregulated in a sustained manner during both acute and chronic equine placentitis and that their activation drives a downstream inflammatory and apoptotic signaling network through TLR4/RAGE and NF-κB-mediated pathways. To test this hypothesis, we combined experimental models with clinical cases to: (1) quantify and localize S100A8 and S100A9 expression in acute and chronic placentitis, and (2) analyze the expression of their known receptors and downstream effectors in clinical placentitis using RNA sequencing (RNA-seq) data. Elucidating this axis is essential for understanding the pathogenesis of placentitis and for evaluating S100A8/A9 as novel diagnostic or therapeutic targets to mitigate pregnancy loss, while also contributing to a broader understanding of alarmin-driven inflammatory pathways across disease contexts.

## 2. Results

### 2.1. mRNA Expression of S100A8 and S100A9 in Equine Chorioallantois (CA) of Acute and Chronic Ascending Placentitis Model

**Figure 1:**
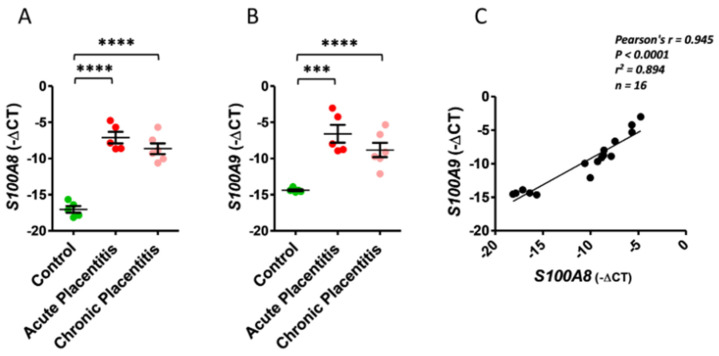
Alterations in calgranulin A *(S100A8)* and B *(S100A9)* mRNA expression in equine chorioallantois (CA) during acute and chronic placentitis models. Quantitative RT-PCR data are expressed as −ΔCT. (**A**) Quantitative changes in *S100A8* during acute and chronic placentitis relative to control mares. (**B**) Quantitative changes in *S100A9* during acute and chronic placentitis relative to control mares. Error bars represent SEM. ***, *p* < 0.001; ****, *p* < 0.0001. (**C**) Pearson`s correlation of *S100A8 and S100A9* mRNA expression levels in equine CA during acute and chronic placentitis. *n* = 5, control; *n* = 5, acute placentitis; *n* = 6, chronic placentitis.

The mRNA expression levels of *S100A8* and *S100A9* were significantly upregulated in chorioallantois (CA) samples retrieved from acute (*p* < 0.001) and chronic placentitis (*p* < 0.0001) groups compared to controls (Figure 1A,B). Conversely, no significant difference in *S100A8* and *S100A9* mRNA expression levels was observed in CA samples between mares with acute and chronic placentitis. A strong positive correlation (*r* = 0.945, *p* < 0.0001) was seen between *S100A8* and *S100A9* expression in all samples (Figure 1C).

### 2.2. Immunolabelling of S100A8 and S100A9 in Equine CA of Acute and Chronic Ascending Placentitis Model

**Figure 2:**
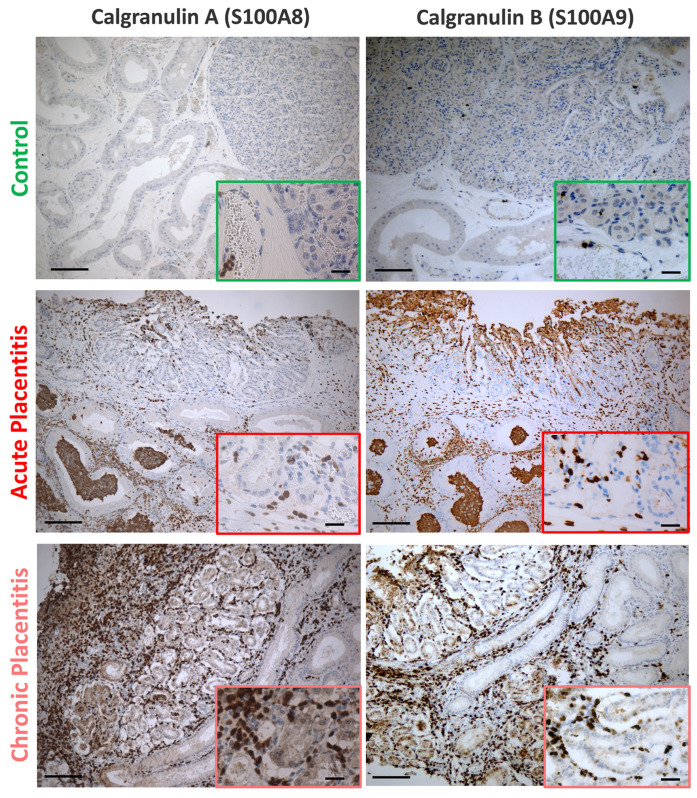
Representative photomicrographs for calgranulin A (S100A8) and B (S100A9) protein expression in equine placenta during acute and chronic placentitis models. Note the increased numbers of cells immunoreactive to S100A8 and S100A9 in placental tissues of mares suffering acute (MIDDLE panels) and chronic (LOWER panels) placentitis compared to those of control mares (UPPER panels). Images are representative and are presented to illustrate protein localization; immunohistochemical staining was not used for quantitative assessment. Scale bar = 100 µm.

Based upon immunolocalization, S100A8 and S100A9 were exclusively expressed in leukocytes (apparently neutrophils and macrophages). A higher number of S100A8/9-positive cells were observed in the CA and endometrium (EN) of placentitis samples (i.e., acute and chronic models) compared to controls (Figure 2). Furthermore, in the placentitis samples, these positive cells had infiltrated the tissue, whereas in control samples, positive immunolabeling was confined mainly to neutrophils and macrophages within blood vessels.

### 2.3. Changes in Gene Expression of S100A8 and S100A9 and Their Downstream Factors in Equine CA During Clinical Ascending Placentitis

**Figure 3:**
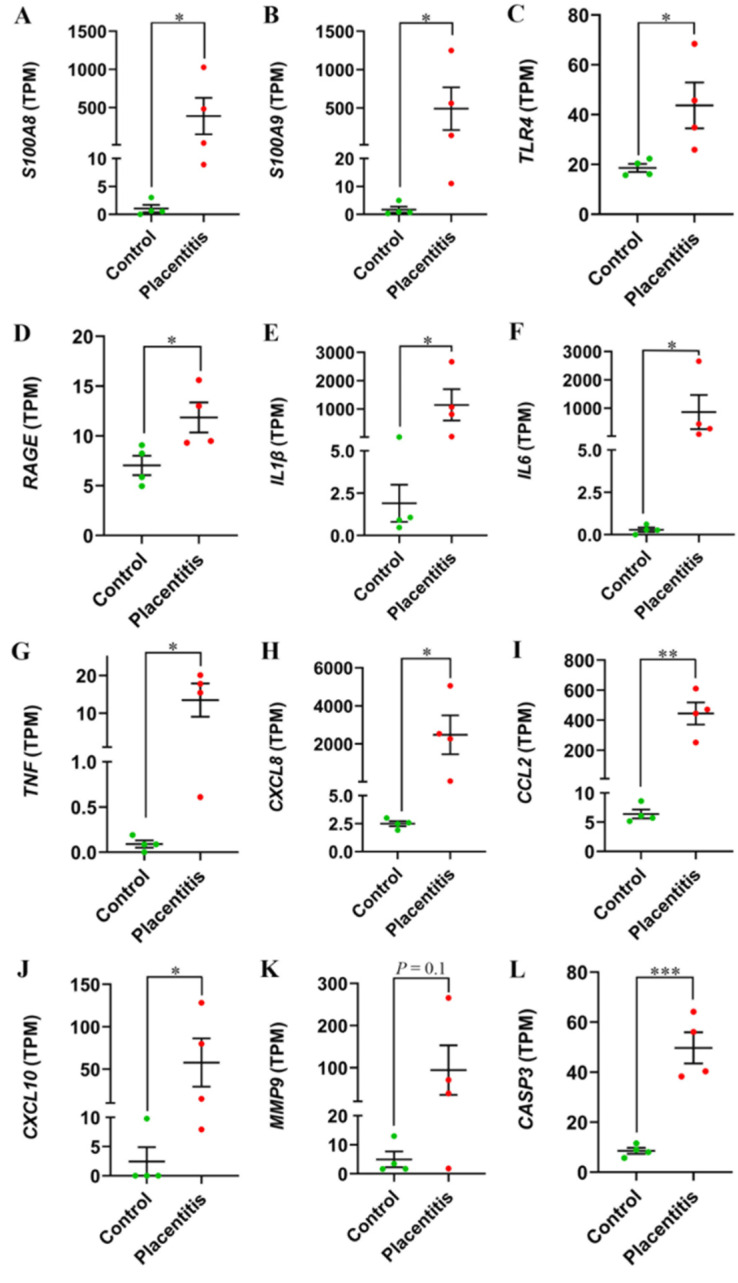
Transcripts per million (TPM) values (shown for visualization) of selected inflammatory targets in equine chorioallantois: clinical ascending placentitis (*n* = 4) versus controls (*n* = 4, normal postpartum placenta). (**A**) Calgranulin A, *S100A8*. (**B**) Calgranulin B, *S100A9*. (**C**) Toll-like receptor 4, *TLR4.* (**D**) Receptor for advanced glycation end products, *RAGE.* (**E**) Interleukin-1β, *IL1β*. (**F**) Interleukin-6, *IL6*. (**G**) Tumor necrosis factor, *TNF*. (**H**) C-X-C motif chemokine ligand 8, *CXCL8*. (**I**) C-C motif chemokine ligand 2, *CCL2*. (**J**) C-X-C motif chemokine ligand 10, *CXCL10*. (**K**) Matrix metalloproteinase 9, *MMP9*. (**L**). Caspase 3, *CASP3.* Each dot represents one mare; bars show mean ± SEM. Differential expression statistics were computed using an unpaired T-test. *, *p* < 0.05; **, *p* < 0.01; ***, *p* < 0.001.

To elucidate the dynamics of S100A8/A9 downstream signaling in clinical placentitis, we compared the expression of ten known downstream factors in CA samples collected from mares with clinical ascending placentitis and healthy controls. The comparison was based on their transcripts per million (TPM) values [23]. Except for *matrix metalloproteinase 9* (*MMP9*; *p* = 0.18, fold change [FC] = 19.2), all investigated downstream factors of *S100A8*/*A9* exhibited significant upregulation during clinical placentitis. These factors included pattern-recognition receptors (*TLR4*; *p* = 0.04, FC = 2.4), immunoglobulin superfamily cell-surface receptors (*RAGE*; *p* = 0.04, FC = 1.7), proinflammatory cytokines (*IL1β*; *p* = 0.04, FC = 948; *IL6*; *p* = 0.03, FC = 1,311; *TNF*; *p* = 0.02, FC = 148; and *CXCL8*/*IL8*; *p* = 0.03, FC = 989), chemokines (C–C motif chemokine ligand 2 [*CCL2*]; *P* = 0.001, FC = 70; and C–X–C motif chemokine ligand 10 [*CXCL10*]; *p* = 0.049, FC = 24), and caspase-family cysteine proteases (caspase 3 [*CASP3*]; *p* = 0.0006, FC = 5.8), as shown in Figure 3.

### 2.4. Immunolabelling of Inflammatory and Apoptotic Markers in Equine CA of Clinical Ascending Placentitis

**Figure 4:**
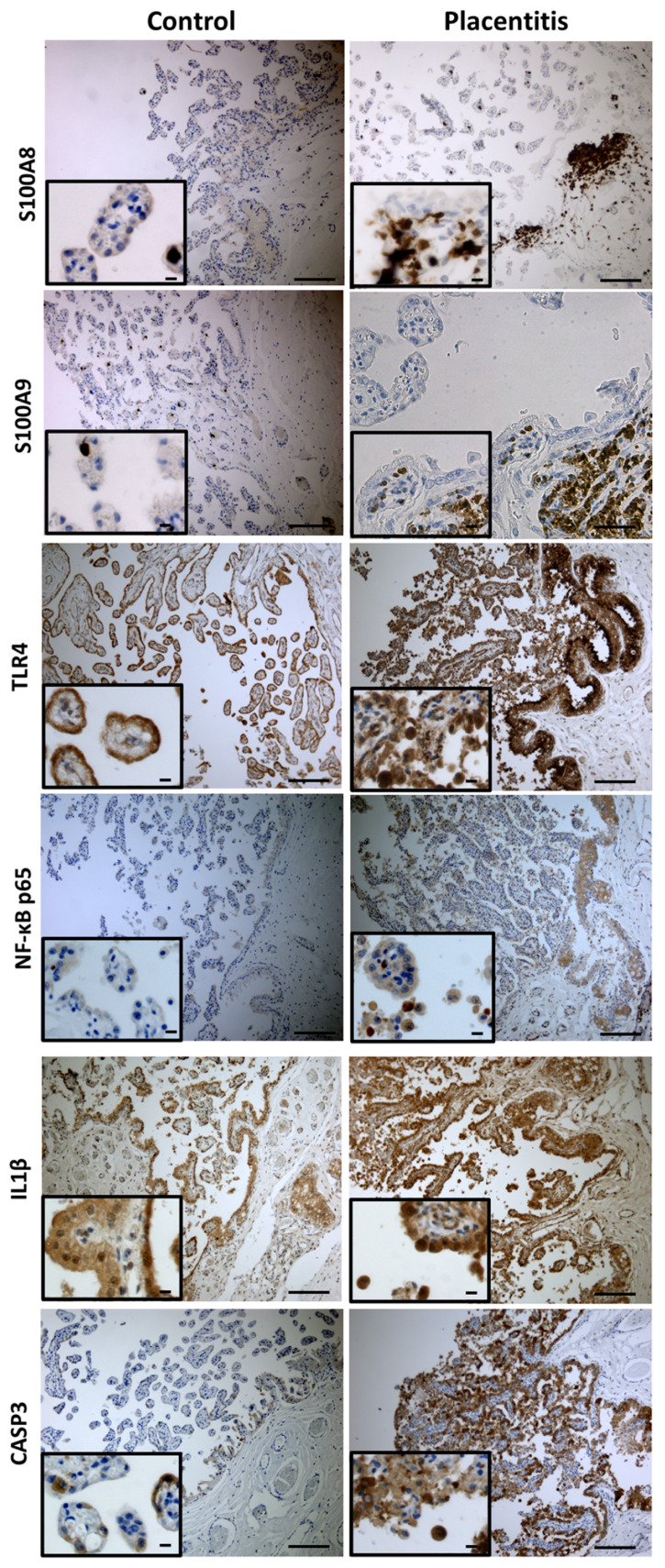
Representative photomicrographs for calgranulin A (S100A8) and B (S100A9), Toll-like receptor 4 (TLR4), Nuclear factor kappa B (NF-κB) p65, Interleukin-1β (IL1β), and Caspase 3 (CASP3) protein expression in equine placenta during clinical ascending placentitis. Mares with placentitis show inflammation and apoptosis. Note the increased number of cells immunoreactive to all inflammatory markers in placental tissues of mares suffering ascending placentitis (RIGHT panels) compared to those of control mares (LEFT panels). Images are representative and are presented to illustrate protein localization; immunohistochemical staining was not used for quantitative assessment. Scale bar = 100 µm.

In the equine chorioallantois, distinct immunolabeling patterns were observed between groups (Figure 4). S100A8 and S100A9 expressions were minimal in controls, limited to rare stromal leukocytes, whereas placentitis showed moderate, patchy staining in stromal macrophages and occasional neutrophils with rare focal trophoblast labeling. In clinical ascending placentitis, both proteins were strongly upregulated, with dense subepithelial accumulations of neutrophils and macrophages and occasional trophoblast positivity. TLR4 exhibited basal staining in control trophoblasts but was markedly increased in placentitis, with enhanced expression in both trophoblasts and infiltrating leukocytes. NF-κB p65 immunolabeling was absent to minimal in controls, with negative trophoblast nuclei, but was markedly upregulated in placentitis, showing intense nuclear and cytoplasmic staining in trophoblasts and infiltrating leukocytes, with prominent nuclear localization in inflammatory cells within the stroma and subepithelial regions. IL1β was detected in controls but showed higher cytoplasmic labeling in inflammatory infiltrates and occasional trophoblasts in placentitis tissues. CASP3 expression was limited in controls to rare trophoblasts or stromal cells, while placentitis samples demonstrated increased staining in trophoblasts, stromal cells, and inflammatory cells, consistent with enhanced apoptosis. Collectively, these findings highlight pronounced activation of inflammatory and apoptotic pathways in CA during placentitis, compared with the quiescent state in controls.

## 3. Discussion

Placentitis remains a predominant cause of late-term pregnancy losses in mares, inflicting substantial losses on the equine breeding industry [24,25,26,27]. The pathophysiology of ascending placentitis involves a complex inflammatory cascade at the maternal–fetal interface, yet the upstream initiators and amplifiers of this response remain incompletely elucidated. Our study confirms the alarmins S100A8 and S100A9 as pivotal upstream mediators that are robustly and persistently upregulated in equine placentitis, and provides mechanistic evidence linking their expression to the activation of a pro-inflammatory and pro-apoptotic signaling network that culminates in placental damage.

A key finding of our study is the significant upregulation of S100A8 and S100A9 mRNA and protein in the CA during both acute and chronic experimentally induced placentitis. The strong positive correlation between their expression levels suggests coordinated regulation, likely as the S100A8/A9 heterodimer (calprotectin), which is known to be the predominant and functionally active form [28]. Notably, this upregulation was not a transient feature of acute infection but was maintained in the chronic phase of the disease. This persistent expression implies a sustained role for these alarmins in driving inflammation, potentially through a positive feedback loop where inflammatory cytokines, such as TNF and IL1β—whose production they stimulate—further enhance S100A8/A9 expression in infiltrating and resident cells. The spatial and cellular context of this upregulation is critical. Immunohistochemistry confirmed that S100A8/A9 expression was predominantly localized to infiltrating neutrophils and macrophages at the site of infection. This aligns with their well-characterized role as DAMPs, which amplify immune cell recruitment and activation [29,30,31,32,33]. The detection of these proteins in leukocytes within placental blood vessels of control mares, in contrast to their extensive tissue infiltration in placentitis, visually underscores the dramatic mobilization of the innate immune system in response to infection. Our findings are consistent with human studies where elevated S100A8/A9 levels in placental tissues and maternal serum are associated with recurrent pregnancy loss and intra-amniotic infection [15,16,17,18,19], suggesting a conserved pathogenic role across species. To decipher the mechanistic consequences of S100A8/A9 upregulation, we leveraged transcriptomic data from clinical cases of ascending placentitis. The concurrent elevation of the known S100A8/A9 receptors, *TLR4* and *RAGE*, provides a plausible pathway for alarmin signaling in the diseased placenta. Activation of these receptors is an established trigger of the NF-κB pathway [34,35]. The marked nuclear translocation of NF-κB p65 in trophoblasts and infiltrating leukocytes in placentitis tissues, as revealed by immunohistochemistry, provides direct protein-level evidence of this pathway’s activation. This cascade culminated in the significant upregulation of a panel of NF-κB-driven effector molecules, including the pro-inflammatory cytokines *IL1β*, *IL6*, and *TNF*, and the potent neutrophil chemoattractant *CXCL8* (IL8). The upregulation of *CCL2* and *CXCL10* further points to a coordinated chemokine response capable of recruiting monocytes and T lymphocytes, respectively, thereby shaping a complex inflammatory milieu [36,37,38,39,40].

Beyond inflammation, our data link S100A8/A9 signaling to pathways directly associated with tissue damage and fetal expulsion. The upregulation of *CASP3* and its increased protein expression in trophoblasts and stromal cells near lesions indicate enhanced apoptosis, a known contributor to placental dysfunction in preterm labor [41,42,43,44]. While *MMP9* did not reach statistical significance in our RNA-seq dataset, a trend toward increased expression was observed. MMP9 hyperactivation in other inflammatory contexts leads to degradation of the extracellular matrix, compromising placental integrity and facilitating membrane rupture [45,46,47,48]. The collective upregulation of these factors paints a coherent picture of how S100A8/A9 activation could contribute to the structural and functional breakdown of the placental barrier.

**Figure 5:**
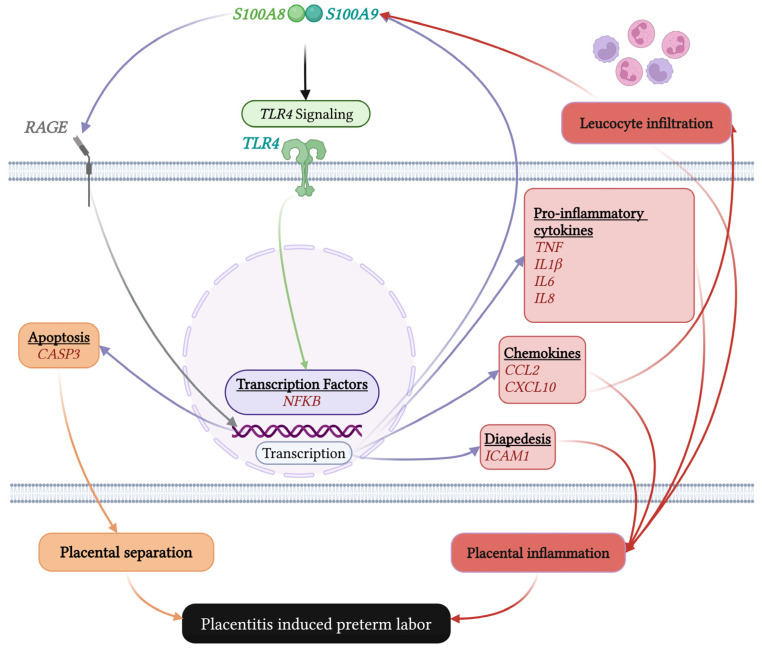
Proposed model of calgranulin A (S100A8) and B (S100A9)-mediated inflammatory signaling in equine ascending placentitis. Ascending bacterial infection triggers recruitment and activation of neutrophils and macrophages at the maternal–fetal interface, leading to release of the alarmins S100A8 and S100A9. These proteins signal through including Toll-like receptor 4 (TLR4) and the receptor for advanced glycation end products (RAGE), on placental and immune cells, resulting in activation of Nuclear factor kappa B (NF-κB). Subsequent induction of pro-inflammatory cytokines, chemokines, and apoptotic mediators drives sustained inflammation, immune cell infiltration, and placental tissue damage, ultimately causing placental separation and insufficiency and culminating in placentitis-induced preterm labor.

A proposed model of S100A8/A9 action in equine placentitis is summarized in Figure 5. Bacterial infection triggers the recruitment and activation of leukocytes, which release S100A8/A9. These alarmins act in an autocrine and paracrine manner via TLR4 and RAGE receptors on placental and immune cells, driving NF-κB activation. This, in turn, initiates a feed-forward loop of pro-inflammatory cytokine and chemokine production, sustained immune cell recruitment, and the induction of apoptotic and tissue-remodeling enzymes, ultimately leading to placental separation and preterm labor. A limitation of this study is the relatively small sample size, reflecting ethical, logistical, and financial constraints inherent to equine reproductive research, which may limit the detection of smaller effect sizes. Nevertheless, the identification of statistically significant differences supports the biological relevance of our model within the context of the current study and provides a foundation for future investigations with larger cohorts when feasible.

Importantly, the mechanistic framework defined here for equine placentitis aligns with a growing body of literature describing S100A8/A9 as a central amplifier of inflammatory pathology across diverse organ systems. In cardiovascular disease, S100A8/A9-driven activation of TLR4 and RAGE promotes endothelial dysfunction, leukocyte recruitment, and sustained NF-κB signaling, contributing to vascular inflammation and tissue injury [13,49]. Similarly, in neurological and oncologic contexts, aberrant S100A8/A9 signaling has been linked to chronic immune activation, disruption of tissue homeostasis, and disease progression [6,50]. The convergence of these pathways with those identified in the equine placenta suggests that S100A8/A9 operates through conserved inflammatory circuits that transcend tissue type, with the maternal–fetal interface representing a uniquely vulnerable but immunologically specialized environment. Thus, placentitis may be viewed not as an isolated reproductive disorder, but as a context-specific manifestation of a broader alarmin-driven inflammatory paradigm, reinforcing the translational relevance of targeting S100A8/A9-mediated signaling in pregnancy-associated as well as systemic inflammatory diseases.

## 4. Materials and Methods

### 4.1. Ethics

All procedures were conducted in accordance with the Institutional Animal Care and Use Committee of the University of Kentucky (Approval Nos. 2013-1190, 2014–1215, 2014-1341, and 2019-3269).

### 4.2. Experimental Design

#### 4.2.1. Expression of S100A8 and S100A9 in CA of Mares with Experimentally Induced Acute and Chronic Ascending Placentitis

Sixteen reproductively normal mares (*Equus caballus*) were enrolled in the present experiment. The animals were housed at the University of Kentucky Maine Chance Farm in pastures and received ad libitum access to food and water. Their age was 4.39 ± 0.18 years (mean ± SEM, range: 4–6 years). To achieve pregnancy, mares were housed with a fertile stallion (4 years old) for 45 days, and all mares became pregnant during this period. Pregnancy diagnosis was confirmed 18–35 days of gestation using transrectal ultrasonography. Gestational age was estimated based on the morphological appearance of the embryos [51].

A total of 16 mares were randomly assigned to the following groups: control (*n* = 5), chronic placentitis (*n* = 6), and acute placentitis (*n* = 5) (Table S2). For the acute ascending model, placentitis was induced in five mares at 280 (275–285) days of gestation via intracervical inoculation with *Streptococcus equi* subsp. *zooepidemicus* (5 × 10^6^ to 25 × 10^6^ CFU in 0.5 mL Todd–Hewitt broth; acute placentitis group) as previously described [52]. For the chronic ascending model, placentitis was induced in six mares at 280 (277–282) days of gestation via intracervical inoculation of *Streptococcus equi* subsp. *zooepidemicus* (1 × 10^6^ CFU in 0.5 mL Todd–Hewitt broth; chronic placentitis group) as previously described [53]. Another five healthy mares with matched gestational age were used as controls (control group). After bacterial inoculation, all mares were monitored daily by transrectal ultrasonography to assess the combined thickness of the uterus and placenta (CTUP) and the degree of placental separation as a measure of disease progression [54]. Mares in the placentitis group were euthanized when they exhibited sufficient signs of the disease (i.e., an increase in CTUP of 3+ mm and/or >50% of the original thickness, and considerable placental separation) [54]. Mares in the control group were also euthanized at corresponding gestational ages. Mares of both groups were humanely euthanized with an overdose of sodium pentobarbital in accordance with the guidelines of the American Veterinary Medical Association for the euthanasia of animals [55]. Mares were euthanized as part of a larger, institutionally approved study requiring collection of the intact gravid uterus and multiple maternal and fetal tissues; euthanasia was not performed solely for the present analyses, and non-terminal sampling approaches were insufficient to meet the broader scientific objectives. Full-thickness placenta (EN and CA) was obtained from the caudal pole of the placenta (cervical star region) [56]. The CA was gently detached from the EN and stored in RNAlater™ (#AM7021; Thermo Fisher Scientific, Waltham, MA, USA) at −80 °C until RNA isolation. Additionally, placental tissues from the same regions were immediately fixed in 10% formalin for 24 h and routinely processed for immunohistochemistry.

#### 4.2.2. Expression of S100A8/A9 and Their Downstream Transcripts in CA of Mares Affected with Clinical Cases of Placentitis

To gain insights into key factors regulated by *S100A8/A9* during equine placentitis, we reanalyzed our previously published RNA-seq data of CA samples that were collected within 3 h of foaling or abortion from mares affected with ascending placentitis (clinical placentitis group, *n* = 4) and normal postpartum placenta (control group, *n* = 4) (Gene Expression Omnibus; GSE186617) [51]. Previous work from our group demonstrated that RNA isolated from chorioallantoic tissue collected within 3 h of foaling consistently exhibited high integrity (RNA integrity number [RIN] > 9) [57]. These findings support the effectiveness of on-farm chorioallantoic tissue collection followed by preservation in RNAlater™ for downstream molecular analyses of the term equine placenta. Bacterial isolation was performed on the placentitis samples and *Streptococcus equi* subsp. *zooepidemicus* was isolated from two cases, while *Escherichia coli* was isolated from the remaining two cases (Table S3).

RNA-seq reads were analyzed using the bioinformatics pipeline as described elsewhere [23]. Briefly, reads were trimmed for quality and adapters with TrimGalore 0.4.4, then mapped to EquCab3.0 using STAR 2.5.2b [58]. Expression values of all transcripts were measured as fragments per kilobase of transcript per million mapped reads, FPKM. All values were quantified using Cufflinks 2.2.1 [59] with the Ensembl annotation (Equus_caballus_Ensembl_95 gtf). The data were normalized as TPM as described elsewhere.

### 4.3. Total RNA Extraction and qRT-PCR

Total RNA was extracted from CA tissue samples using RNeasy Mini Kit (#74104; Qiagen, Germantown, MD, USA) and reverse-transcribed into complementary DNA using TaqMan™ Reverse Transcription Reagents (#4368814; Thermo Fisher Scientific) as previously shown [53,60]. qRT-PCR was performed in duplicate using PowerUp™ SYBR™ Green Master Mix (#A25741; Thermo Fisher Scientific). The primer sequences used are listed in Table S1. Actin beta (*ACTB*) and glucuronidase beta (*GUSB*) were set as reference genes [60,61]. Delta CT (ΔCT) values were calculated via subtraction of the geometric mean of the CT values of the reference gene from the CT value of the gene of interest.

### 4.4. Immunohistochemistry

Formalin-fixed placental samples were processed for paraffin embedding using standard histological techniques. Five-μm-thick sections were cut, mounted on glass slides, and dried overnight at 37 °C. Immunohistochemical staining of paraffin sections for S100A8 [23,53,62], S100A9 [53,62], CASP3, TLR4 [63], NF-κB p65, and IL1β was performed using a Leica BOND-MAX system (Leica Microsystems, Buffalo Grove, IL, USA) as previously described [62,63]. For immunohistochemistry of the tissue sections, we employed a recombinant mouse anti-S100A8 monoclonal antibody (1:1000; #sc-48352, Santa Cruz Biotechnology, Inc., Dallas, TX, USA), a mouse anti-S100A9 monoclonal antibody (1:1000; #MA1-81381, Thermo Fisher Scientific), a mouse anti-CASP3 monoclonal antibody (1:50; #sc-271759, Santa Cruz Biotechnology, Inc.), a rabbit anti-IL1β polyclonal antibody (1:50; #P420B, Thermo Fisher Scientific), a mouse anti-NF-κB p65 (A-12) monoclonal antibody (1:100; #sc-514451; Santa Cruz Biotechnology, Inc.), and rabbit anti-TLR4 polyclonal antibody (1:200; #PA5-23284, Thermo Fisher Scientific). Before incubation with the primary antibody, the tissue sections were subjected to heat-induced epitope retrieval using an EDTA-based pH 9.0 epitope retrieval solution for 20 min (S100A8, S100A9, IL1β, NF-κB) or using a citrate-based pH 6.0 epitope retrieval solution for 20 min (CASP3, TLR4). CASP3, NF-κB p65, and IL1β were validated using spleen and kidney equine tissues (Figure S1). Negative controls omitting the primary antibody, using the EDTA-based or citrate-based retrieval methods, are shown in Figure S1. Immunohistochemistry was performed to assess cellular and tissue localization of target proteins; staining was evaluated qualitatively, and no quantitative comparisons between groups were performed.

### 4.5. Data Analysis

The qRT-PCR gene expression results were shown as −ΔCT [64]. Differences in mRNA expression of *S100A8* and *S100A9* between groups were analyzed using one-way ANOVA followed by Tukey’s multiple comparisons of means. Pearson’s correlation was employed to evaluate the association between *S100A8* and *S100A9* mRNA expressions using GraphPad Prism version 10.0.0 for Windows (GraphPad Software, Boston, MA, USA)

The RNA-seq results are presented as TPM, as described [65]. An unpaired T-test was used to evaluate the significance of changes in mRNA expression of *S100A8*/*A9* and transcripts involved in their downstream molecular pathways between the control and clinical placentitis groups. Although gestational ages were tightly clustered within late gestation, exact matching between all control and placentitis cases was not achieved, which represents a limitation that should be addressed in future studies with larger cohorts.

No formal a priori power analysis was conducted. Sample size was constrained by ethical considerations, animal availability, and the substantial financial and logistical demands inherent to equine reproductive research, which reflect common challenges in large-animal studies. Sample size was therefore determined based on feasibility while aiming to maximize experimental value.

Differences of *p* < 0.05 were considered statistically significant, and any difference of 0.1 > *p* ≥ 0.05 was considered a trend. Descriptive statistics are expressed as the mean ± standard error of the mean (SEM).

## 5. Conclusions

In conclusion, our data firmly establishes S100A8 and S100A9 as critical upstream regulators in the inflammatory cascade of equine placentitis. Their persistent expression from acute to chronic stages and their strong correlation with a defined downstream network of inflammatory and tissue-destructive mediators highlight their potential as both diagnostic biomarkers and therapeutic targets. Future studies should focus on quantifying S100A8/A9 in maternal plasma to evaluate its utility as a non-invasive early diagnostic tool for placentitis. Furthermore, investigating therapeutic strategies to disrupt the S100A8/A9 signaling axis, perhaps through targeted inhibition of TLR4 or RAGE, could offer a novel approach to mitigate the devastating pregnancy loss associated with this condition.

## Data Availability

RNA-seq data are publicly available in the NCBI Gene Expression Omnibus (GEO) under accession number GSE186617. Other supporting data are available in Tables S2 and S3.

## Supplementary Materials

The following supporting information can be downloaded at: https://www.mdpi.com/article/10.3390/ijms27031550/s1.

## Abbreviations

The following abbreviations are used in this manuscript:

CA	Chorioallantois
CASP3	Caspase 3
CLC2	Chemokine (C-C motif) ligand 2
CXCL8	Chemokine (C-X-C motif) ligand 8
CXCL10	Chemokine (C-X-C motif) ligand 10
DAMPs	DA.M.A.ge-associated molecular pattern molecules
IL1β	Interleukin-1β
IL6	Interleukin-6
IL8	Interleukin-8
NF-κB	Nuclear factor kappa B
qRT-PCR	Quantitative RT-PCR
PRRs	Pattern recognition receptors
RAGE	Receptor for advanced glycation end products
RNA-seq	RNA sequencing
S100A8	Calgranulin A
S100A9	Calgranulin B, or calprotectin
TLR	Toll-like receptor
TLR4	Toll-like receptor 4
TNF	Tumor necrosis factor
TPM	Transcripts per million
