# Supplementary material for: Activation of the S100A8/A9 Alarmin Amplifies Inflammatory Pathways in Equine Ascending Placentitis
Source: Int J Mol Sci. 2026 Feb 4;27(3):1550. doi: 10.3390/ijms27031550 (PMC12897833; doi:10.3390/ijms27031550)
Supplement: Supplementary file 1 [file ijms-27-01550-s001.zip › ijms-4051037-Figure S1.pdf]

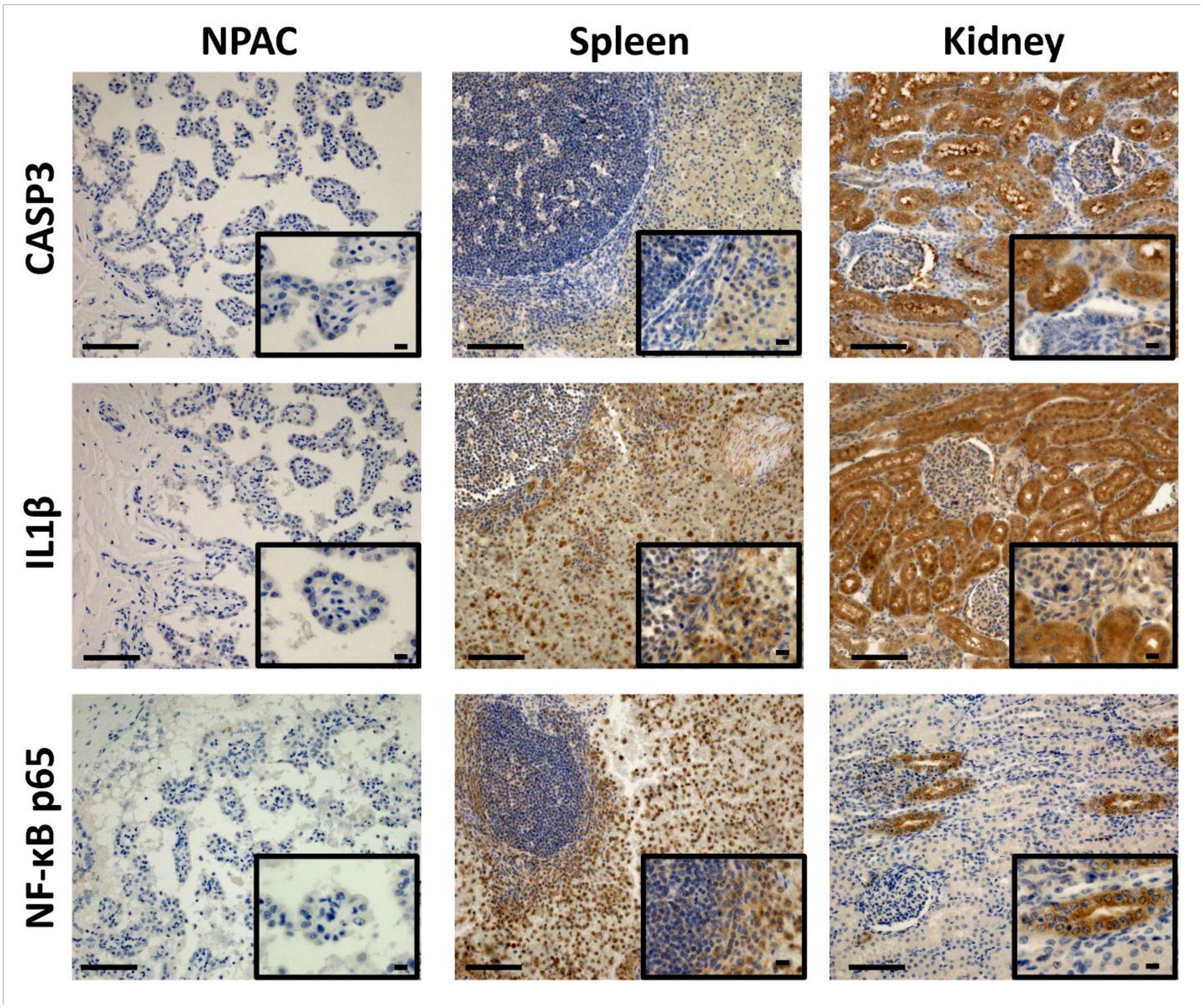

Figure S1. Validation of antibody specificity and negative controls for immunohistochemistry. Representative images demonstrate the absence of signal following omission of the primary antibody (NPAC in equine placenta, citrate-based pH 6.0 epitope retrieval solution for 20 min, TOP PANEL, and EDTA-based pH 9.0 epitope retrieval solution for 20 min, MIDDLE AND BOTTOM PANELS) and confirm the expected staining patterns in equine spleen (positive control) and kidney (tissue specificity control), thereby validating antibody specificity for Caspase 3 (CASP3), Nuclear factor kappa B (NF-κB) p65, and Interleukin-1β (IL1β) protein expression in equine tissues. Scale bar = 100 μm.
