# Supplementary material for: Activation of the S100A8/A9 Alarmin Amplifies Inflammatory Pathways in Equine Ascending Placentitis
Source: Int J Mol Sci. 2026 Feb 4;27(3):1550. doi: 10.3390/ijms27031550 (PMC12897833; doi:10.3390/ijms27031550)
Supplement: Supplementary file 1 [file ijms-27-01550-s001.zip › ijms-4051037-Table S1. S100A8_S100A9.pdf]

Table S1. Primers used in the present study.

Gene	Sequence (5′–3′)	Accession ID	Product Length (bp)
S100A8	Forward: TGCTTTATGGGCTACCAGGG<br>Reverse: GAGTTGGACTTCACTGCGGA	XM_001494358.4	*87*
S100A9	Forward: CATGCTCGTGCATCTTCTCAT<br>Reverse: CAGCTGAGTTTCGAGGAG-TTCA	XM_001494378.5	*79*
ACTB	Forward: CGACATCCGTAAGGACCTGT<br>Reverse: CAGGGCTGTGATCTCCTTCT	NM_001081838	*100*
GUSB	Forward: GGGATTCGCACTGTGGCTGTCA<br>Reverse: CCAGTCAAAGCCCTTCCCTCGGA	XM_001493514	*117*

ACTB, actin beta; GUSB, glucuronidase beta; *S100A8,* S100 calcium-binding protein A8; *S100A9,* S100 calcium-binding protein A9.
